# Luteolin inhibits migration of human glioblastoma U-87 MG and T98G cells through downregulation of Cdc42 expression and PI3K/AKT activity

**DOI:** 10.1007/s11033-013-2632-1

**Published:** 2013-05-16

**Authors:** Wen-Yu Cheng, Ming-Tsang Chiao, Yea-Jiuen Liang, Yi-Chin Yang, Chiung-Chyi Shen, Chiou-Ying Yang

**Affiliations:** 1 Institute of Molecular Biology, National Chung Hsing University, 250 Kuo Kuang Road, Taichung, 402 Taiwan; 2 Department of Neurosurgery, Taichung Veterans General Hospital, Taichung, Taiwan; 3 Department of Medicine, National Defense Medical Center, Taipei, Taiwan; 4 Tri-Service General Hospital, National Defense Medical Center, Taipei, Taiwan; 5 Department of Physical Therapy, Hungkuang University, Taichung, Taiwan

**Keywords:** Glioblastoma, Migration, Luteolin, PI3K/AKT, Cdc42, Proteasome degradation

## Abstract

Luteolin (3′,4′,5,7-tetrahydroxyflavone) is a common flavonoid in many types of plants and has several beneficial biological effects, including anti-inflammation, anti-oxidant, and anti-cancer properties. However, the detail mechanisms of luteolin in suppressing tumor invasion and metastasis are poorly understood. Here, we investigated the effects of luteolin on suppressing glioblastoma tumor cell invasion and migration activity. Under the non-cytotoxic doses (15 and 30 μM), luteolin exhibited an inhibitory effect on migration and invasion in U-87 MG and T98G glioblastoma cells. Additionally, filopodia assembly in U-87 MG cells was markedly suppressed after luteolin treatment. The treatment of luteolin also showed a decrease of Cdc42 (cell division cycle 42) protein levels and reduced PI3K/AKT activation, whereas there was no association between this decrease and phosphorylated ERK or altered transcription levels of Cdc42. Over expression of constitutive Cdc42 (Q61L) using transient transfection in U-87 MG cells induced a partial cell migration, but did not affected the degradation of the protein levels of Cdc42 after luteolin treatment. Moreover, inhibition of the proteaosome pathway by MG132 caused a significant recovery in the migration ability of U-87 MG cells and augmented the Cdc42 protein levels after luteolin treatment, suggesting that pharmacological inhibition of migration via luteolin treatment is likely to preferentially facilitate the protein degradation of Cdc42. Taken together, the study demonstrated that flavonoids of luteolin prevent the migration of glioblastoma cells by affecting PI3K/AKT activation, modulating the protein expression of Cdc42 and facilitating their degradation via the proteaosome pathway.

## Introduction

Glioblastoma multiforme (GBM), the most common and most aggressive primary brain tumor, is a highly malignant lesion with poor prognosis. The standard therapeutic strategy for GBM is multimodality treatment, including surgical resection and postoperative radiotherapy combined with chemotherapy. Whereas, more than 70 % of GBM patients die within 2 years of diagnosis [1]. In high-grade glioma, the results of contemporary drug treatments are not satisfactory. Fatal GBMs are characterized by rapid cell proliferation and aggressive invasion and destroy of the surrounding normal brain tissue.

Luteolin is one of the most common flavonoids presents in edible plants and in plants are used to treat a wide variety of pathologies by traditional medicine. The potential benefits of luteolin (50 mg/kg body weight) in CNS include decreased inflammation and axonal damage by preventing monocyte migration across the blood–brain barrier (BBB) [2]. Preclinical studies have shown that this flavones possesses a variety of pharmacological activities, including anti-inflammation, anti-oxidation, anti-proliferation anti-angiogenesis, and anti-metastasis [2–13]. In prostate cancer, the investigators found that luteolin suppressed proliferation and induced apoptosis in vitro and in vivo via inhibition of the IGF/1R/AKT signaling pathway [14]. Bagli et al. [15] have proved that luteolin inhibited tumor growth and angiogenesis in a murine xenograft model and decreased vascular endothelial growth factor (VEGF)-induced in vivo angiogenesis via inhibition of the phosphatidylinositol 3′-kinase (PI3K) pathway. Other reports show that luteolin inhibits and poisons topo I and II in leukemia cells [16] and Chinese hamster ovary AA8 cells [17]. The activity of luteolin on topoisomerases I and II may has therapeutic implications, as these enzymes are the target of several drugs such as etoposide, topoteca, irinotecan, commonly used in the treatment of cancer. However, another evidence suggest that luteolin may prevent these processes by inhibiting matrix metalloproteinases (MMPs) and focal adhesion kinase (FAK) [18]. Luteolin plays an anti-tumorigenic role in various cancers through inhibiting carcinogen activation, suppressing cell growth, inducing cell apoptosis and inhibiting metastasis [19]. However, the detail mechanisms of luteolin-mediated inhibition of glioblastoma cell migration are, yet, unclear.

The Ras-ERK1/2 MAP kinase pathway plays a critical role in numerous cellular processes, including proliferation, differentiation, survival, and motility [20]. Growth factors activate ERK-MAPK by signaling through their cognate receptors at the cell surface to the small GTPase Ras. ERK activity may also be induced via ECM signaling during adhesion. In this pathway, the small GTPases Rac and cell division cycle 42 (Cdc42) activate PAK (p21-activated kinase), which phosphorylates MEK to make it a more efficient Raf substrate [21]. Whereas at Rho family gene, Cdc42 is an important for cell motility and able to induce a mesenchymal-amoeboid transition in melanoma cells [22]. Cdc42 GTPase is a key signaling component governing actin cytoskeleton organization, adhesion, migration, proliferation, and survival in mammalian cells [23]. Active forms of Rac1 and Cdc42 regulated the direction of cell movement and have a positive effect on E-cadherin mediated cell–cell adhesions which increased number of filopodia, actin reach finger-like protrusions [24]. By inhibiting the activation of Cdc42 showed the importance in reducing motility and invasion in glioma cells.

In this study, we proved that luteolin inhibited the migratory and invasion in human glioblastoma U-87 MG and T98G cells. The migration property of luteolin-mediated inhibition was exerted via inhibition of phosphorated PI3K/AKT and resulted in rapid Cdc42 proteolysis.

## Materials and methods

### Chemicals

Luteolin (>98 % purified) was obtained from Cayman Chemical Company (Cat 10004161). Stock solutions of luteolin were prepared in DMSO and stored at −20 °C. Cycloeximide (CHX) (#C4859) and MG132 (Z-Leu-Leu–Leu-al) (#C2211) from Sigma-Aldrich (USA). Subsequent dilutions were made in Dulbecco’s modified Eagle’s medium (DMEM).

### Cell culture and transfection

Human glioblastoma cell line (U-87 MG and T98G), and HUVECs used in this study was obtained from the American type culture collection (ATCC, Manassas, VA). U-87 MG and T98G cells were maintained in monolayer culture in DMEM (GIBCO, Rockville, MD, USA), containing 10 % fetal bovine serum (FBS) (Hyclone, Logan, UT), 100 μg/ml penicillin and 100 μg/ml streptomycin (Gibco BRL, Rockville, MD) at 37 °C in a humidified atmosphere comprising of 95 % air and 5 % CO_2_. HUVECs grown in Clonetics Endothelial Cell Growth Medium-2 (EGM-2; Lonza, Walkersville, MD). Transient transfected U-87 MG cells were generated by transfection with 1 μg of pEGFP-C1 or pcDNA3-EGFP-Cdc42-Q61L (purchased from Addgen: plasmid 12986) using jetPEI (Polyplus-transfection, 101-10) and incubated for 48 h. The medium was removed and fresh medium containing 30 μM of luteolin was added to the wells, then treated continuously with luteolin for 24 h.

### Cell viability assay

Cells were seeded in 96-well plates at a density of 1 × 10^4^ per well for 24 h and then treated with various concentrations of luteolin (dissolved in 0.1 % dimethyl sulfoxide (DMSO and diluted with DMEM medium with 4 % FBS to 10, 20, 30, 40, 50 μM) for 24 h. The DMSO in culture medium did not exceed 0.1 %, a concentration known not to affect cell viability. Cell viability was determined by 3-(4,5-dimethylthiazol-2-yl)-5-(3-Carboxymethoxyphenol)-2-(4-Sulfophenyl)-2H-tetrazolium, inner salt (MTS) method, following the manual of Cell Titer 96 Aqueous One Solution Cell viability assay (Promega, Cat.#: G3582). The cell viability in each group was determined by absorbance under an optical density value of 490 nm (450–540 nm) with a 96-well plate reader.

### Wound healing assay

In a 24-well plate, U-87 MG and T98G cells were added to high glucose DMEM media, and incubated for 24 h in order to create a monolayer of cells. A scratch was made in the middle of the well with a P200 pipette tip. The debris was washed away and new media was added to the wells. Under the micro-scope, the cells were imaged and the initial area of the scratch for the field of view was determined by multiplying the length by the average width of the area devoid of cells. The plate was incubated at 37 °C for 12 h, after which the same field of view was imaged and the area devoid of cells was recalculated by the same method.

### Invasion and migration assay

The invasion assay was performed by using 24-well BD Biocoat Matrigel invasion chambers with 8 μm polycarbonated filters (Becton–Dickinson, Bedford, MA). U-87 MG cells were seeded on Matrigel invasion chamber plates, and cultured in routine medium. Cells were incubated for 24 h at 37 °C in a humidified incubator with 5 % CO_2_. Nonmigratory cells on the upper surface of the filter were removed by wiping with a cotton swab. Invasive cells that penetrated through pores and migrated to the underside of the membrane were stained with Giemsa solution after fixation with 4 % formaldehyde in PBS. The cell number was counted under microscopic vision, and the average cell number was determined. The migration assay was similar to invasion assay. Overall, the migration assay is similar to the invasion assay except for that it does not contain Matrigel chambers.

### RT-PCR assay

The expression of Cdc42 gene was quantified by reverse transcriptase PCR. Total RNA was prepared from the cell line using RNeasy Mini kit (Qiagen, Inc.) For cDNA preparation, 1 mg of total RNA was retro transcribed by High-Capacity cDNA Archive Kit (Applied Biosystems, Foster City, CA, USA) at 37 °C for 2 h. PCR amplification was performed in a 50 μl reaction volume containing 1 μg cDNA reaction and using ampliTaq Gold polymerase (Perkin Elmer). The primer sequences used for each PCR are outlined below. Primer sequences for Cdc42 were (F): 5′-TATGATTGGTGGAGAACCAT-3′ and (R): 5′-ATTCTTTAGGCCTTTCTGTG-3′ (Genbank accession no. M57298, resulting in a 370 bp PCR product).

### Western blot analysis

Cells were lysed in lysis buffer containing 50 mM Tris–HCl, pH 7.5, 150 mM NaCl, 1 % Nonidet P-40, 0.25 % sodium dexycholate, 0.1 % SDS with complete protease inhibitor cocktail (Roche, 04693159001) and protein concentration was assayed with Bio-Rad Protein Assay Kit (Bio-Rad, 500-0006). Equal amounts of proteins from each sample were separated by sodium dodecyl sulfate (SDS) polyacrylamide gel electrophoresis and transferred to polyvinylidene difluoride membrane (PVDF) (Amersham, RPN303F). Membrane was blocked for 1 h in TBS containing 5 % non fat milk and 0.2 % Tween 20. For the detection of ERK, phospho-ERK, Cdc42, Rac1 and GAPDH. For the detection of antibodies : ERK (Santa Cruz, sc-93), phospho-ERK (Santa Cruz, sc-7383), phospho-PI3 kinase p85 (Cell Signaling, #4228), phospho-SAPK/JNK (Cell Signaling, #4668), phosphor-p38 (Cell Signaling, #4511), F-actin (Novus, NB100-64792), Cdc42 (Santa Cruz, sc-8401), Rac1 (Santa Cruz, sc-95), anti-GFP (Novus, SP3005P), phosphor-AKT (pS473) (Epitomics, #2118), AKT (Epitomics, #1080), phosphor-FAK (Novus, NB100-92712) and GAPDH (Santa Cruz, sc-25778), these primary antibodies were incubated with membranes at 4 °C overnight. Finally, the membranes were incubated with horseradish peroxidase-conjugated secondary antibody and developed using ECL Western blot reagents (PerkinElmer, nel104).

### Phalloidin stain of filopodia

Filopodia of U-87 MG cells were detected using fluorescent phalloidin and analyzed by confocal microscopy. Cells were seeded to the glass cover slips in cell dishes (24 well plate) overnight. Luteolin was then added and cells were cultured for 24 h. After fixing with 4 % paraformaldehyde, cells were treated with 0.1 % Triton X-100 for 20 min and blocked with 3 % BSA for 30 min. Then cells were incubated with 100 nM Rhodamine-conjugated phalloidin for 30 min and examined under confocal laser scanning microscope (1200× oil). The results were repeated at three independent experiments in each case.

### Statistical 1analysis

Multiple samples were collected in each measurement and expressed as mean ± standard deviation. Single-factor analysis of variance (ANOVA) method was used to assess the statistical significance of the results. *p* values < 0.05 or 0.01 were considered statistically significant.

## Results

### Effects of luteolin treatment in U-87 MG and T98G glioblastoma cells

**Fig. 1:**
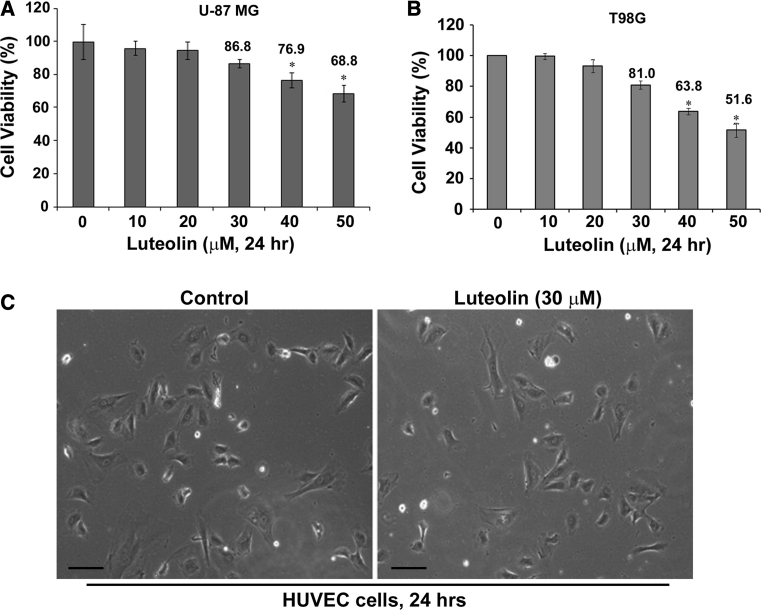
Effect of luteolin in U-87 MG and T98G cells viability. **a** U-87 MG cells and **b** T98G cells were seeded in 96-well plates at a density of 1 × 10^4^ per well and incubation O/N, and then treated with various concentrations (0, 10, 20, 30, 40 and 50 μM) of luteolin for 24 h. Cell viability was measured by MTS assay. Values are the mean ± SEM results from three independent experiments. *p < 0.05. **c** Effect of luteolin (30 μM) on HUVECs viability in culture after the treatment with DOSM (*left panel*) or luteolin for 24 h. Cell death of the HUEVCs was observed by light microscopy. *Scale bars* 50 μm

We investigated whether luteolin administration increases the cellular cytotoxicity and to look for the appropriate concentration of luteolin for further study in the possible novel physiological effects. U-87 MG and T98G glioblastoma cells were treated with various concentrations (0, 10, 20, 30, 40 and 50 μM) of luteolin for 24 h and examined the effects of luteolin on different glioblastoma cell lines were examined with MTS assay. As shown in Fig. 1, the cell viability was no significant decreased at conc. up to 20 μM of luteolin, even conc. up to 30 μM of luteolin (retained 86.8 % of viability cells). However, the declines of the U-87 MG cell population might represent the increases of dead cells when the concentration of luteolin is more than 40 μM (23.1 %) (Fig. 1a). Similar cytotoxicity effects of luteolin were observed in T98G cells (Fig. 1b). In addition, we showed that in addition to no inhibiting growth of the glioblastoma cells, luteolin (30 μM) decreased cancer cell migration but do not affect normal cell growth (human umbilical vein endothelial cell, HUVECs) (Fig. 1c). Therefore, to exclude the cytotoxic effects of excess luteolin, the following experiment would to use 30 μM of luteolin to determine the related effects on U-87 MG and T98G cells.

### Luteolin inhibits migration and invasion in U-87 MG and T98G cells

**Fig. 2:**
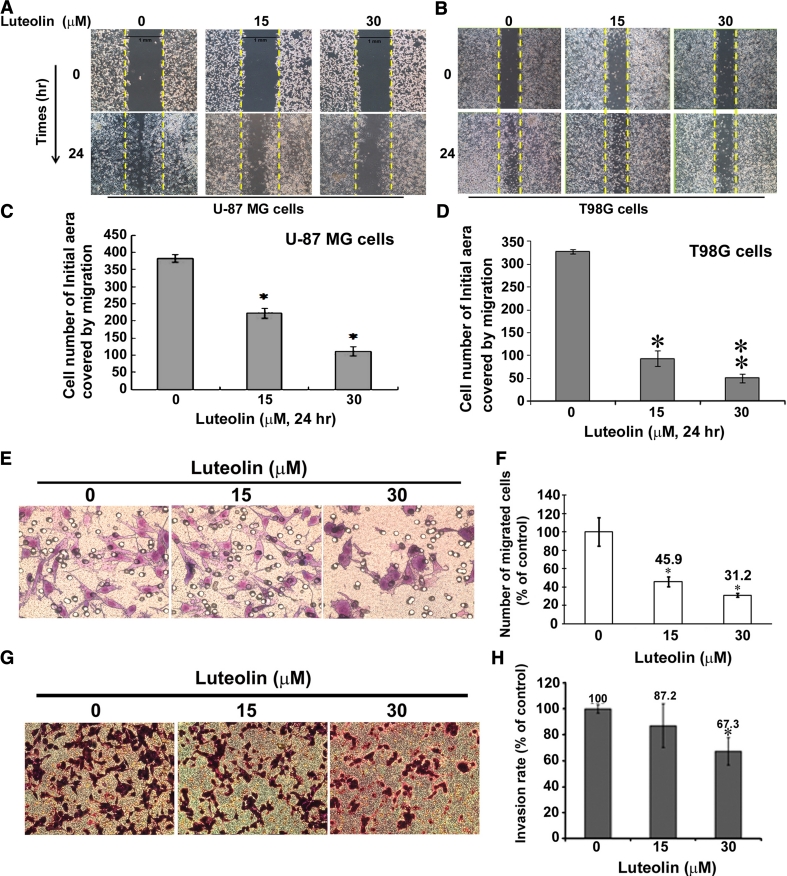
Luteolin inhibited migration and invasion of U-87 MG and T98G cells. Wound healing assay was performed on **a** U-87 MG cells and **b** T98G cells. Cells (1.2 × 10^5^ cells/24 well) were treated with luteolin (0, 15, and 30 μM) for 24 h. The plates were photographed at 0 and 24 h post-wounding. The cells migrating into the wound area were counted based on the *dashed line* as time zero. **c,** **d** The quantification analysis of above wounds healing quantification. Data are expressed as the mean ± SE from three independent experiments. *p < 0.05. **p < 0.01. **e** Boyden chamber for evaluation of migration in U-87 MG cells treated with luteolin. **f** Quantification of migration in Boyden chamber. **g** Invasion chamber assay for evaluating the invasion rate of U-87 MG cells treated with luteolin. The bottom chamber was filled with DMEM medium. The upper chambers of membrane were coated with 3 μg of Matrigel. After luteolin treatment for 24 h, the invading U-87 MG cells passed through the membrane and were quantified by counting the cells that migrated onto the membrane. Cells were fixed, stained, and counted as described in the text. **h** Quantification of invasion in Invasion chamber system. Invasion rate of 100 % is defined by control group which contained invasive U-87 MG cells without treatment of luteolin. The data are presented the as mean ± SD results from triplicate independent experiments. Indicates significantly difference from vehicle of U-87 MG and *p < 0.05

GBM patients dies more often from invasion and migration, therefore cell migration is a key feature of tumor progression and malignant. To investigate the anti-migratory effects of luteolin, U-87 MG cells were examined using wound healing assay. The monolayer of U-87 MG and T98G cells was scratched with a pipette tip and treated with moderated concentrations of luteolin (0, 15, and 30 μM) for 24 h. As shown in Fig. 2a, b, there is a significant drop in the ability of the luteolin 30 and 15 μM treated cells to migrate into the empty space compared with vehicle control in U-87 MG and T98G cells (Fig. 2c, d). The Boyden chamber assay, originally introduced by Boyden for the analysis of leukocyte chemotaxis, is based on a chamber of two medium-filled compartments separated by a microporous membrane. We therefore determined the effect of luteolin on cellular migration in U-87 MG glioblastoma cells. As shown in Fig. 2e, U-87 MG cells treatment with luteolin resulted in a significantly inhibition of cell migration, as well as shown in Fig. 2f present at 15 μM (45.9 %) and 30 μM (31.2 %) under luteolin treatment. To further determine the effects of luteolin on the cell invasive properties of the previously established cell models, using penetration through addition of Matrigel™ in above method as measured invasion ability. U-87 MG cells was performed for evaluating the ability of cellular invasion. As shown in Fig. 2g, the number of cells crossed Matrigel in the luteolin treatment was significant decreased compared to the control cells, as shown in Fig. 2h, U-87 MG cells was slightly decreased at luteolin at 15 μM (87.2 %) and 30 μM (67.3 %), respectively. These results suggested that although luteolin (30 μM) treatment did not significantly affect the cellular viability, the luteolin inhibited the cell migration and invasion in U-87 MG and T98G cells.

### Luteolin inhibits migration of glioma cells through down-regulation of Cdc42 expression

**Fig. 3:**
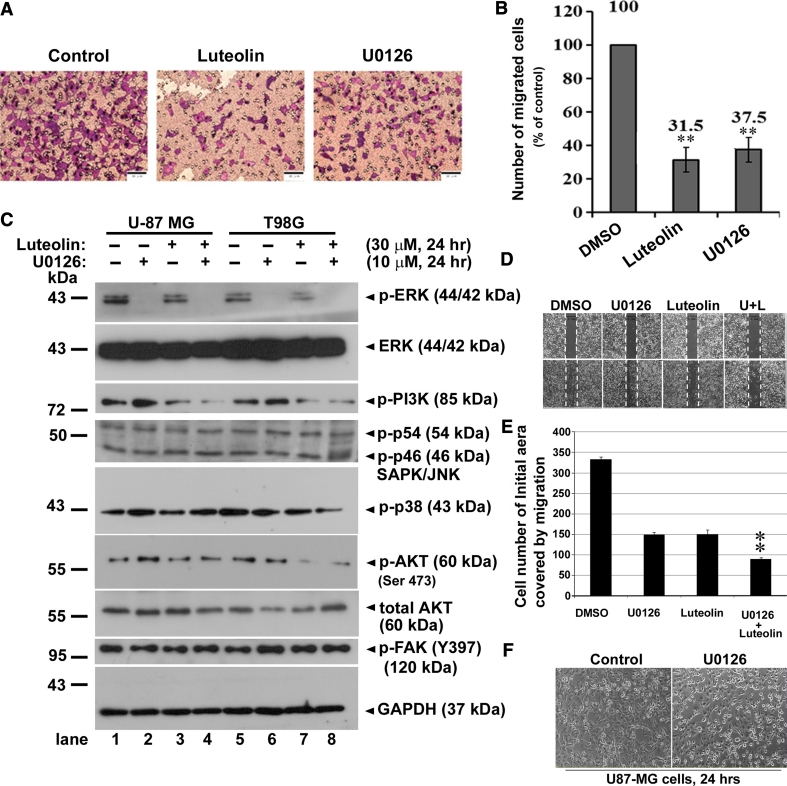
Luteolin and U0126 inhibited the migration of U-87 MG cells. **a** Boyden chamber assay for evaluation of migration in glioma cells treated with luteolin (30 μM) or specific ERK inhibitor U0126 (30 μM) for 24 h. **b** Quantification of migration was modified by Boyden chamber assay. Scale bars are 50 μm. Data were expressed as the mean ± SE from three independent experiments. **p < 0.001. **c** U-87 MG and T98G cells were treated for 24 h with luteolin (30 μM), followed by western blot analysis for phosphorylated ERK (p-ERK), total ERK, p-PI3 K, p-JNK, p-p38, p-AKT, total AKT and FAK. GAPDH was a loading control. These results are representative of three independent experiments. **d** Wound healing assay was performed on U-87 MG cells. Luteolin (L) (30 μM) with or without 10 μM U0126 (U) were added. Migrated cells were quantified by manual counting. **e** The above data were presented the quantification analysis of above wounds healing quantification. Data are expressed as the mean ± SE from three independent experiments. **p < 0.01 compared with control group. **f** Light-microscopy of U-87 MG cells were treated without/with U0126 (30 μM) for 24 h

We sought to investigate the cause of the inhibition of cell migration upon luteolin treatment in U-87 MG cells. We noted that ERK1/2 was inactivated using the MEK inhibitor, U0126, the cell migration was significantly inhibited (Fig. 3a) as previously study [25]. The cell migration in the luteolin treatment of U-87 MG cells was significantly inhibited using Boyden chamber assay detection (Fig. 3a). The quantification of migratory ability of U-87 MG cells treated with U0126 or luteolin was significant decreased to 37.5 and 31.5 %, respectively. (Fig. 3b). Therefore, to examine the change in the activation of ERK and other key regulatory pathways signal pathway after the luteolin treatment, whole cell lysate of U-87 MG cells after treatment with luteolin for 24 h were analyzed for protein expression of phosphor-ERK, total ERK, using Western Blotting assay. As shown in Fig. 3c, the protein expressions of phosphorylated ERK1/2 (p-ERK) were significantly reduced after U0126 treatment. However, the protein expression of p-ERK was not significantly affected by luteolin, and the expression of total ERK also remained consistent, indicating that there was no association between luteolin treatment and any inhibition of migration ability in the active ERK pathway. Remarkably, both PI3 K and AKT activations were partially reduced in the intensity of phosphorylation protein after luteolin treatment, whereas other key regulatory pathways, such as p38, FAK and JNK, did not affected, suggesting that luteolin could reduced PI3K/AKT activation in glioblastoma cells (Fig. 3c). To evaluated the function effect of combination of U0126 and luteolin to inhibit cell migration, we performed the wound-healing assay in U-87 MG cells. As shown in Fig. 3d, the single treatment of U0126 or luteolin have a significantly similar inhibitory of migration, and cotreatment showed a synergy inhibitory effect of migration in U-87 MG cells (Fig. 3e). In addition, we also examined the cellular cytotoxicity of U0126 at 30 μΜ concentration and did not significantly affected in the growth of U-87 MG cells (Fig. 3f). Taken together, these data indicate that the inhibitory of migration in luteolin is likely mediated through impaction on PI3K/AKT activation.

**Fig. 4:**
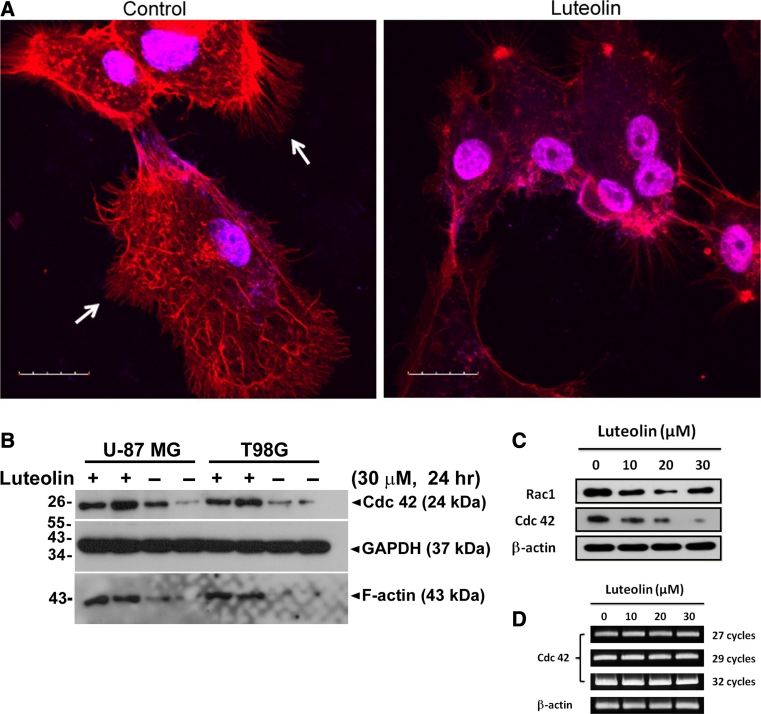
Luteolin disrupts actin assembly and filopodia formation. **a** Glioblastoma cells U-87 MG were assessed by fluorescence staining with Rhodamine phalloidin, a substance which specifically detects F-actin. In the control, filopodia are present (*white arrow, left*). Incubation of U-87 MG cells with luteolin (30 μM) for 24 h led to marked disruption of filopodia assembly (right). *Scare bars* are 20 μm. **b** U-87 MG and T98G cells were treated with luteolin (30 μm) for 24 h, followed by western blot analysis for Cdc42 and F-actin. GAPDH was a loading control. **c** U-87 MG cells were treated with 0, 10, 20, 30 μm concentration of luteolin for 24 h, followed by western blot analysis for Cdc42 and Rac1. β-actin was a loading control. **d** Total mRNA were concentrated in U-87 MG cell after luteolin (0, 10, 20 and 30 μM) for 24 h and gene expression levels of Cdc42 were assessed by RT-PCR

Several studies have shown that actin polymerization, which leads to filopodia assembly and depolymerization, play a crucial role in cell motility [26, 27]. We attempted to explore the molecular events in response to treatment with luteolin, the formation of the actin cytoskeleton in U-87 MG cell was assessed by staining with Rhodamine-phalloidin, which specifically binded F-actin [28]. The results showed that filopodia assembly in U-87 MG was abolished by luteolin (30 μM) (Fig. 4a). Therefore, luteolin suppressed the migration of U-87 MG at least in part disrupting actin assembly and filopodia formation.

Cell division cycle 42 signaling is essential for cell motility and is related to filopodia formation. To further explore luteolin effects on the cell cytoskeleton with regard to oncogenic transformation, we examined the protein expression levels of Cdc42 in U-87 MG and T98G cells. This analysis indicated that added luteolin (30 μM) attenuated the protein levels of Cdc42 in U-87 MG and T98G cells as well as the expression of the F-actin protein (Fig. 4b). The protein expression levels of Cdc42 exhibited at a dose-dependent decrease upon luteolin treatment, whereas the protein levels of Rac1 were decreased to a lesser extent (Fig. 4c). Notably, the mRNA levels of Cdc42 were not impacted after luteolin treatment of U-87 MG cells (Fig. 4d), suggesting that there was no association between the decrease of Cdc42 protein levels and the levels of mRNA transcription.

To determine whether the effect of luteolin reduced the half-life of Cdc42 protein, we examined effects of cycloheximide (CHX), a protein synthesis inhibitor. U-87 MG cells were treated with 50 μg/ml CHX to block new protein synthesis. Cdc42 proteins in the present of luteolin showed more rapid degradation kinetics compared to the control treatment (Fig. 4e). The half-life of Cdc42 in the present of luteolin was approximately 24 h, whereas the half-life of co-treatment with luteolin and CHX has been reduced to less than 8 h (Fig. 4e). These results demonstrated that luteolin resulted in rapid Cdc42 proteolysis in glioblastoma cells.

**Fig. 5:**
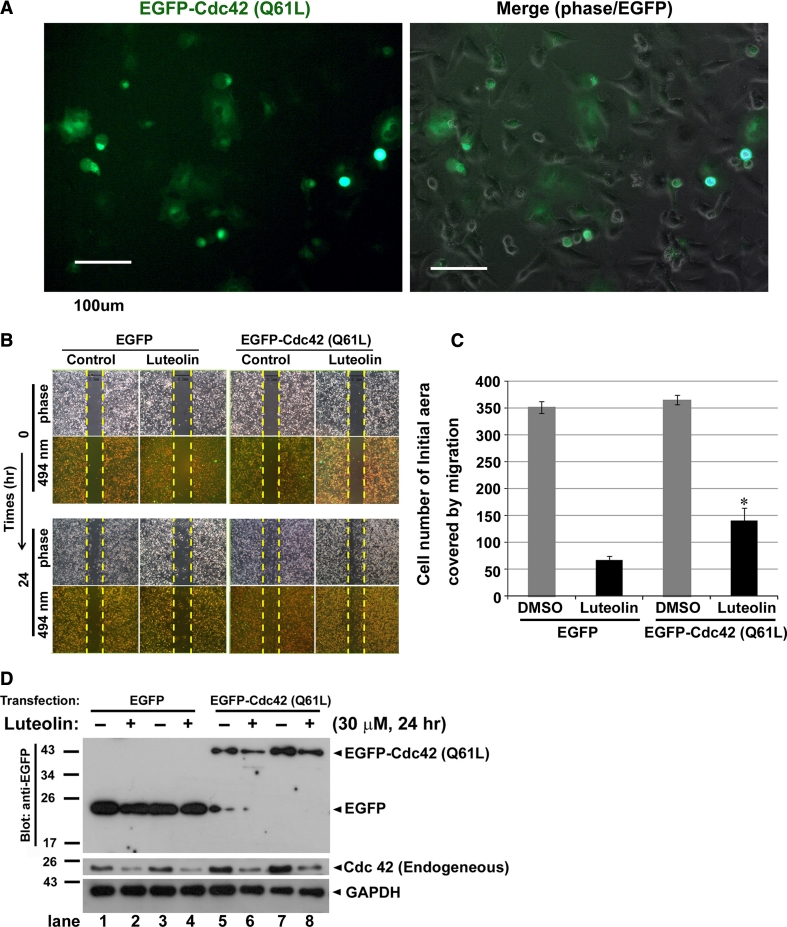
Effect of transfected constitutive active Cdc42 (Q61L) in U-87 MG cells upon luteolin treatment. **a** phenotypes of U-87 MG cells transfected with EGFP fused to constitutive active mutant of Cdc42 (Q61L) (*green flurescence*) and merged with phase image; *bar represents* 100 μm. **b** Wound healing assay was performed on U-87 MG cells after transfected with EGFP or EGFP-Cdc42 (Q61L). Luteolin (30 μM) were added to U-87 MG cells. Migrated cells were quantified by manual counting. **c** The above data were presented the quantification analysis of above wounds healing quantification. Data are expressed as the mean ± SE from three independent experiments. *p < 0.05 compared with luteolin group of transfected with EGFP control plasmid DNA. **d** After transfected with EGFP or EGFP-Cdc42 (Q61L), U-87 MG cells were treated with luteolin (30 μΜ) for 24 h, followed by western blot analysis for anti-Cdc42 and anti-GFP. GAPDH was a loading control

To explore the importance of Cdc42 activation in the induced cell migration observed, transient transfection of constitutive active Cdc42 (EGFP-Cdc42-Q61L) in U-87 MG cells was performed (Fig. 5a). Significant green fluorescence was observed in U-87 MG cells (approximately 30–35 % of total cells; Fig. 5a). Subsequent analysis of the wound healing properties showed that moderate Cdc42 activation increased a partial cell migration response upon luteolin treatment (Fig. 5b, c). Notably, the protein levels of Cdc42 were substantially impaired both exogenously and endogenously after luteolin treatment (Fig. 5d), further illustrating that the mechanism of luteolin in inhibiting migration potentially facilitated the degradation of the Cdc42 protein levels.

**Fig. 6:**
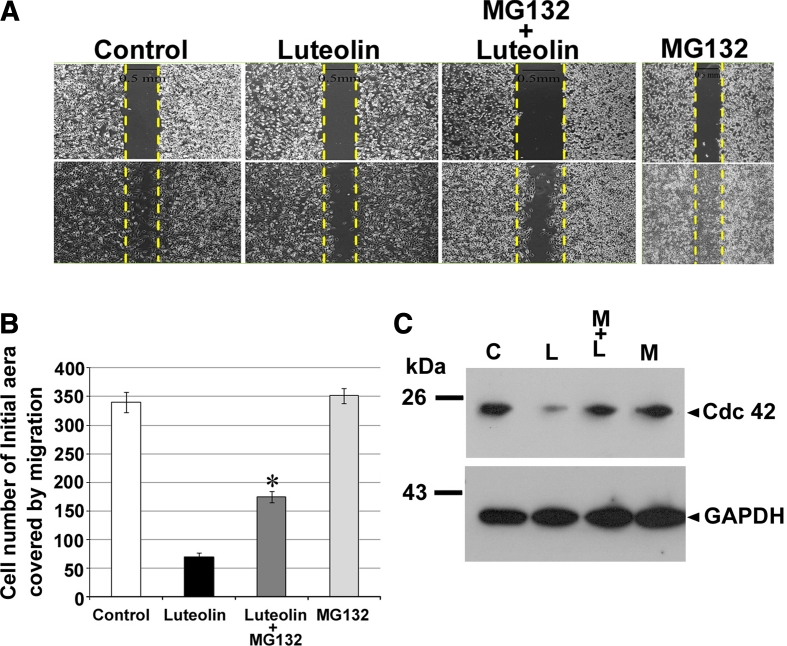
The inhibitory of migration by luteolin treatment was restored by MG132 treatment. **a** Wound healing assay was performed on U-87 MG cells. Luteolin (30 μM) with or without 0.125 μM MG132 were added to U-87 MG cells. MG132 was added 4 h before counting cells. Migrated cells were quantified by manual counting. **b** The above data were presented the quantification analysis of above wounds healing quantification. Data were expressed as the mean ± SE from three independent experiments. *p < 0.05 compared with luteolin group. **c** U-87 MG cells were treated with luteolin (30 μΜ) for 24 h with or without 0.125 μM MG132 were added. MG132 was added 4 h before collecting cells, followed by western blot analysis for Cdc42. GAPDH was a loading control

To determine whether the inhibitory effect of luteolin on migration in U-87 MG cells was dependent on proteosome degradation of Cdc42, we examined the single and combined effects of luteolin and MG132, a proteasome inhibitor, on U-87 MG cells. As shown in Fig. 6a, following the addition in combination of MG132 (0.125 μM) and luteolin at 4 h before analysis, the number of moving cells were noticeably increased as compared to luteolin only treatment (Fig. 6b), whereas MG132 did not affect the ability of U-87 MG cells to migrate compared to control in vitro. More specifically, MG132 provided a prevention to against the degradation of Cdc42 by luteolin treatment in U-87 MG cells, some similar to control group (Fig. 6c). Thus, the inhibition of migration resulting from the pharmacological mechanism of luteolin is likely to facilitate the protein degradation of Cdc42 by activation of the proteosome degradation pathway.

## Discussion

In this study, we have shown that luteolin at non-toxic contractions is a potent modulator of migration and invasion of U-87 MG and T98G glioblastoma cells. However, we found that luteolin treatment did not interrupt the phosphorylation of ERK as well as affected to a lesser extent on other key regulatory pathway such as MAPK-p38, FAK, and JNK, whereas possible due to luteolin produced the reduction of PI3K/AKT activation. In additionally, we clearly showed that luteolin disrupted cell migration at least in part through preventing filopodia formation and Cdc42 protein levels after luteolin treatment. Transient transfection of constitutive active Cdc42 (EGFP-Cdc42-Q61L) in U-87 MG cells increased the partially ability of migration in the present of luteolin, but did not affect the degradation levels of Cdc42 protein. Finally, we found that luteolin facilitated a significant protein degradation of Cdc42 via the proteaosome-dependent degradation pathway. Collectively, luteolin treatment inhibited the activation of PI3K and thereby suppressed the AKT mediated migration signal pathways, and resulted in rapid Cdc42 proteolysis.

Cell migration events are determined largely by cytoskeleton, the internal scaffolding of proteins that give cell shape, polarity and the capacity to move. Inhibition of U-87 MG and T98G cell motility by luteolin was confirmed by wound healing (Fig. 2a, b, c, d) and modified Boyden chamber assay (Fig. 2e, f, g, d). However, luteolin has no cytotoxic effect on glioma cells at concentrations below 30 μM (Fig. 1). Therefore, we suggested the blockade of migration by luteolin is not through its cytotoxic effects. ERK 1/2 and MAPK subfamily promote the proliferation and motility of cells. Rac1 and Cdc42, which lead to form membrane ruffles/lamellipodia and filopodia respectively, have been shown to regulate a vast spectrum of biological functions, including cell cytoskeleton function and cell polarity during migration [24, 29]. The effects of Cdc42, Rac1 and Rho on the cytoskeleton and cellular adhesion suggest a possible role for Rho proteins in cellular migration [30, 31]. The decrease of Cdc42 and Rac1 may involve in the blocking of cell migration. We found that luteolin inhibited the cell migration of glioblastoma and managed to decrease the protein expression of Cdc42 and F-actin (Fig. 4b). We also observed that luteolin inhibited U-87 MG cell morphology (less spread) using Boyden chamber assay (Fig. 2e, g). The morphology of U-87 MG cells was in response to Cdc42 and Rac1 activity by luteolin treatment.

It is known that the increase in ERK signaling might be highly correlated with GBM proliferation [32] and tumor metastasis. However, the prevention of tumor invasion/migration is one of the goals for glioma patients. The migratory process requires the coordinated activation and targeting of both structural and signaling molecules [33]. Increased expression of ERK signaling might contribute to GBM proliferation [32]. Cytoskeleton changing regulated by Rac1 and Cdc42 correlated with MAPK/ERK signaling pathway may serve critical molecular signaling in the development of cell mobility [30, 34]. Previous studies demonstrated that luteolin inhibited inflammatory responses and down-regulation of ERK signal transduction pathway in human colon epithelial cells and migration of F9 parietal endoderm cells was regulated by the ERK pathway [35]. In our study, luteolin was not affected the phosphorylated ERK as well as other key regulatory pathways such as MAPK-p38, JNK, and FAK (Fig. 3c). However, luteolin showed a particularly inhibitory effect on PI3K and AKT activation, which functional mediator in cell migration [36]. The inhibitory effect of luteolin on PI3K/AKT activity can vary depending on the cellular context. In addition, AKT is downstream serine/threonine kinase in the RTK/PTEN/PI3K pathway and large scale genomic analysis of GBM has demonstrated that this pathway is mutated in the majority of GBMs. This RTK/PTEN/PI3K pathway leads to activated AKT and phosphor-AKT levels are elevated in the majority of GBM tumor samples and cell lines, which studies show help glioma cells grow uncontrolled, evade apoptosis, and enhance tumor migration and invasion. Thus, luteolin targeting the inhibitory of AKT activation represent a potential treatment option against GBM and additional research efforts are required to fully explore and develop this possible treatment strategy.

Although in recent years, the anti-tumorigenic role of luteolin has been well recognized, the role of luteolin in cancer cell migration and progression is poorly understood. Most cancer patients die of metastasis instead of the primary lesion [37, 38]. How to prevent cancer cell migration has became an area of intense research [39, 40]. Infiltration of glioma instead of the primary lesion is always the cause of death for patients diagnosed with malignant gliomas patients which is highly related to the migration and invasion malignant cells, is one of the tough questions for increasing the survival rate. The results of Rac1, Cdc42 and ERK in U-87 MG cells treated with U0126 indicated that the inhibition effects of luteolin in migration and invasion of U-87 MG cells is caused by inhibition of the ERK signal pathway. Luteolin is beneficial in inhibition of glioma cell migration and invasion, which may have good therapy potential in the treatment of brain tumors. A number of studies have shown that stimulation of luteolin resulted in inhibition of migration through down regulation of PDGFR-β, PKC, ERK, Src, PI3 K, and AKT signaling pathway [41–45]. However, our results showed that luteolin partially reduced PI3K/AKT activation in U-87 MG and T98G cells. The effects of luteolin inhibitory AKT activity can vary depending on the cellular context. Interesting, the rapid degradation of Cdc42 was observed by luteolin treatment, even exogenously constitutive active Cdc42 also presented at a significantly decreased by luteolin. By contrast, we showed that the Cdc42 proteins with luteolin treatment exhibit increased degradation in a oroteasome-dependent pathway, as treatment with the proteasome inhibitor MG132 enhanced Cdc42 protein levels. Consequently, a novel luteolin-mediated inhibitory of migration pathway is proposed that luteolin inhibited the invasion and migration of glioblastoma cells is likely to inhibite PI3K/AKT activation and facilitate protein degradation of Cdc42 via the proteaosome degradation pathway. The molecular mechanism underlying this inhibition needs further investigation.

In conclusion, luteolin is able to disrupt the invasion and migration of U-87 MG and T98G glioblastoma cells. The pharmacological mechanism of luteolin that inhibits migration of glioblastoma cells is likely to inhibite PI3K/AKT activation and facilitates protein degradation of Cdc42 via the proteaosome degradation pathway. Our study suggested that the role of luteolin is a potential anti-neoplastic agent in preventing migration and invasion, and provide the well-recognized role of being an effective chemotherapeutic agent for the treatment of GBMs.
